# O08 Neurosarcoidosis mimicking CNS lymphoma

**DOI:** 10.1093/rap/rkaa053.007

**Published:** 2020-11-03

**Authors:** Tarunya Arun

**Affiliations:** University Hospitals of Coventry and Warwickshire, Coventry, United Kingdom

## Abstract

**Case report - Introduction:**

Sarcoidosis is a multisystem disease which involves formation of inflammatory lesions known as granulomas. Central nervous system’s involvement is rare. Clinical neurologic complications occur in approximately 5% of patients.

Diagnostic criteria for neurosarcoidosis in the absence of central nervous system (CNS) histology are not firmly established. A clinically compatible picture, exclusion of other neurological diseases, and histological confirmation of disease elsewhere are generally required.

We present a case report of neurosarcoidosis presenting as a lymphoma mimic.

**Case report - Case description:**

A 45-year-old right-handed white male with past medical history of obstructive sleep apnoea, presented to the acute neurology clinic with several weeks’ history of cognitive decline and severe L facial pain. He had lost 2 stone in weight and there was loss of appetite over 2 months. Neurology examination (including cranial nerves) was unremarkable except for a mini mental state score of 25/30, where he lost points on the attention and recall tasks. CT head revealed a mass in the L cavernous sinus. Brain MRI with contrast revealed an enhancing lesion in the left. suspicious of lymphoma. Additional work up included whole body FDG-PET/CT scan, lumbar puncture. Lumbar puncture showed normal CSF. Serum ACE was normal and a paraneoplastic panel. Whole body PET/CT scan showed FDG avid areas in the bilateral neck, axillary regions, chest and pelvis and inguinal regions, highly consistent with lymphoma. Bone marrow biopsy was negative for lymphoma. Further EBUS biopsy before start of prednisolone revealed multiple non caseating granulomas, diagnostic of sarcoidosis. The patient was treated with oral prednisolone, followed by anti-tumour necrosis factor-a infliximab infusion.

A repeat brain MRI with contrast done at five months after initiation of steroids, methotrexate and infliximab showed complete resolution of the intracranial lesion. Neurological and neuropsychological evaluation three months after diagnosis demonstrated resolution of facial pain and cognitive decline.

**Case report - Discussion:**

There exists several mimics of neurosarcoidosis. Both clinically and radiographically, neurosarcoidosis can be difficult to diagnose. MRI and PET scan in neurosarcoidosis can often mimic malignancy. Early symptomatic treatment is advised for neurosarcoidosis, thus there is a clear need for more prompt diagnosis to allow commencement of the appropriate therapy.

There is no known cure for neurosarcoidosis. Immunosuppression is the primary means of controlling the disease, and corticosteroids are the cornerstone of therapy. Treatment options are limited; however, there is more evidence suggesting that steroids and immunomodulatory agents such as infliximab may improve clinical outcomes, which may be due to the anti-TNF-α effect on reducing oxidative stress.

**Case report - Key learning points:**

Our patient had a clinical presentation suspicious of lymphoma, however he did not have lymphoma and had a good response to corticosteroids and infliximab. Often, FDG PET/CT scan can be misleading and may appear to be neoplastic rather than inflammatory. ACE levels in both CSF and serum are not always positive. Biopsy in these cases is necessary to establish correct diagnosis. Prompt treatment can lead to significant reduction in mortality and morbidity

